# *Cajanus platycarpus* Flavonoid 3′5′ Hydroxylase_2 (*CpF3′5′H_2*) Confers Resistance to *Helicoverpa armigera* by Modulating Total Polyphenols and Flavonoids in Transgenic Tobacco

**DOI:** 10.3390/ijms24021755

**Published:** 2023-01-16

**Authors:** Shaily Tyagi, Maniraj Rathinam, Narasimham Dokka, Nidhee Chaudhary, Lakkakula Satish, Prasanta K. Dash, Ajit Kumar Shasany, Rohini Sreevathsa

**Affiliations:** 1 ICAR-National Institute for Plant Biotechnology, Pusa Campus, New Delhi 110012, India; 2 Centre for Biotechnology and Biochemical Engineering, Amity Institute of Biotechnology, Amity University, Noida 201313, India; 3 Applied Phycology and Biotechnology Division, Marine Algal Research Station, CSIR—Central Salt and Marine Chemicals Research Institute, Mandapam 623519, India

**Keywords:** flavonoids, *Helicoverpa armigera*, herbivory, pigeonpea, polyphenols, ROS scavenging, wild relative

## Abstract

Pod borer *Helicoverpa armigera*, a polyphagus herbivorous pest, tremendously incurs crop damage in economically important crops. This necessitates the identification and utility of novel genes for the control of the herbivore. The present study deals with the characterization of a *flavonoid 3′5′ hydroxylase_2* (*F3′5′H_2*) from a pigeonpea wild relative *Cajanus platycarpus*, possessing a robust chemical resistance response to *H. armigera*. Though *F3′5′H_2* displayed a dynamic expression pattern in both *C. platycarpus* (Cp) and the cultivated pigeonpea, *Cajanus cajan (*Cc*)* during continued herbivory, *CpF3′5′H_2* showed a 4.6-fold increase vis a vis 3-fold in *CcF3′5′H_2*. Despite similar gene copy numbers in the two *Cajanus* spp., interesting genic and promoter sequence changes highlighted the stress responsiveness of *CpF3′5′H_2*. The relevance of *CpF3′5′H_2* in *H. armigera* resistance was further validated in *CpF3′5′H_2*-overexpressed transgenic tobacco based on reduced leaf damage and increased larval mortality through an in vitro bioassay. As exciting maiden clues, *CpF3′5′H_2* deterred herbivory in transgenic tobacco by increasing total flavonoids, polyphenols and reactive oxygen species (ROS) scavenging capacity. To the best of our knowledge, this is a maiden attempt ascertaining the role of *F3′5′H_2* gene in the management of *H. armigera.* These interesting leads suggest the potential of this pivotal branch-point gene in biotic stress management programs.

## 1. Introduction

Pigeonpea, commonly known as red gram, is an ancient and important legume crop cultivated in India [1]. The seeds are not only abundant in essential amino acids such as lysine, threonine, cysteine and arginine, but also in iron and iodine [2]. Hence, it is a predominant dietary protein source for the vegetarian population in the country and plays a significant role in food security. As in the case of any other crop, pigeonpea is threatened by numerous biotic and abiotic stress factors during various stages of growth [3]. *Helicoverpa armigera* or pod borer is one of the biotic stress factors responsible for major crop losses [4] leading to a reduction of yield potential in pigeonpea. Biotechnological interventions to tackle the devastating herbivore and management of yield gap in pigeonpea have been an appropriate endeavor [5,6]. Furthermore, the unavailability of pod borer-resistant sources in the cultivated germplasm, ability of the pest to develop resistance against insecticides and Cry proteins instigated the search for novel genes and approaches for herbivore management [7].

Crop wild relatives have emerged as predominant players in several crop improvement programmes due to an amplitude of resistance traits bestowed to them [8,9]. Many pigeonpea wild relatives have been identified to possess a range of important traits, including pod borer resistance [10]. Our group has been focusing on deciphering the molecular basis of pod borer resistance in one of the pigeonpea wild relatives, *Cajanus platycarpus* [11,12]. The utility of multiomics approaches to study the underlying resistance divulged a strong chemical response [11,13,14] along with other multi-layered strategies in the wild relative to herbivory.

Plants respond to the attack by herbivorous insects through an amalgamation of several intricate molecular processes that include physical, chemical and molecular events. Chemical responses to feeding herbivores that include, signaling, transduction and production of secondary metabolites [3,15] have been one of the prime ways plants react. Secondary metabolites involved in plant–herbivore interactions are products of the phenylpropanoid pathway and comprise flavonoids viz., flavanones, flavonols, flavanols, and anthocyanins. Flavonoids are a group of multiuse low molecular weight secondary metabolites that have proven to play vital roles in various plant physiological activities and defence against biotic and abiotic stresses [11,16,17]. During the assessment of pod borer resistance response in *C. platycarpus,* it was observed that there was a structured reprogramming of flavonoid biosynthesis pathway leading to the hyper-accumulation of several flavonoids [11]. It was also observed that there was synchrony in the overexpression of pivotal genes of the pathway including *chalcone synthase* (*CHS*), *dihydroflavonol 4-reductase* (*DFR*), *flavonoid 3′5′-hydroxylase_2* (*F3′5′H_2*), *flavonol synthase* (*FLS*), *leucoanthocyanidin reductase* (*LAR*), *anthocyanidin synthase* (*LDOX/ANS*) and production of their concomitant metabolites [11]. Understanding the role of these important enzymes in pod borer resistance would create avenues for their utility in the management of this devastating herbivore. This prompted us to characterize and assess *flavonoid 3′5′ hydroxylase* (*CpF3′5′H_2*) an important branch-point enzyme in the production of flavonoids from *C. platycarpus*.

*F3′5′H* belongs to cytochrome P450 family and plays a vital role in the flavonoid biosynthesis pathway [18] as it has a cardinal involvement in the production of anthocyanins and flavan-3-ols. Studies have demonstrated the industrial utility of *F3′5′H* for improving colour in ornamental flowers [19,20]. Additionally, the overexpression of *F3′5′H* has also shown increased flavonoids in many plant species [21]. Further, there have also been several studies establishing the role of other pivotal enzymes of the flavonoid pathway in the management of abiotic stresses [22]. However, no study has been conducted to date elucidating the role of *F3′5′H* overexpression in pod borer management.

Therefore, the primary aim of this study was to characterize *CpF3′5′H_2* from pigeonpea wild relative *C. platycarpus* and assess its role in the management of *H. armigera* in a model plant, tobacco. Information stemming from the study can form a base for the inclusion of a novel plant-based gene in several crop improvement programmes targeting pod borer management.

## 2. Results and Discussion

**Figure 1:**
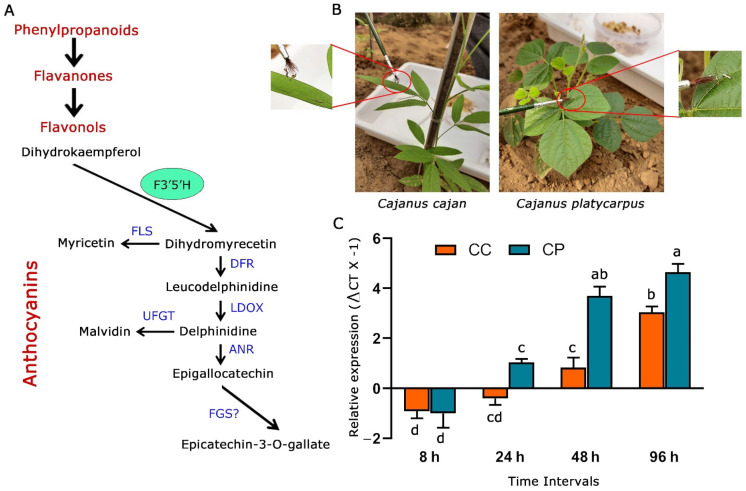
Response of *F3′5′H_2* from *C. cajan* and *C. platycarpus* to herbivory by *H. armigera.* (**A**) General phenylpropanoid pathway highlighting the role *F3′5′H* in anthocyanin biosynthesis. Blue-coloured fonts denote genes responsible for specific metabolite production. F3′5′H: flavonoid 3′5′-hydroxylase, FLS: flavonol synthase, DFR: dihydroflavonol 4-reductase, LDOX: anthocyanidin synthase, UFGT: UDP flavonoid glycosyltransferase, ANR: anthocyanidin reductase, FGS: flavan-3-ol gallate synthase (**B**) Experimental set-up and larval challenge in 45 days old *C. cajan* and *C. platycarpus* plants as standardized earlier [13] in the nethouse. (**C**) Dynamic expression of *F3′5′H_2* gene in both the *Cajanus* spp. challenged by *H. armigera* at different time intervals [a–d represents significance at *p* ≤ 0.05 obtained from two-way ANOVA post hoc Tukey’s test].

Secondary metabolites have been found to play a phenomenal role in various plant activities, including interactions with the environment [23]. The involvement of flavonoids produced by the phenylpropanoid pathway in abetting plants to manage biotic and abiotic stresses is known. However, the in-depth characterization of genes from the flavonoid biosynthesis pathway and their role in deterring herbivores is an exciting area to probe into. The present study was hence envisaged to understand the relevance of *flavonoid 3′5′hydroxylase_2* (*CpF3′5′H_2*) (Figure 1A) during the response of *C. platycarpus* to herbivory by *H. armigera.*

### 2.1. F3′5′H_2 Gene was Upregulated at Varying Levels in C. cajan and C. platycarpus in Response to H. armigera

We initially wanted to assess the response of *F3′5′H_2* to herbivory in the pigeonpea wild relative vis a vis the cultivated counterpart. To determine the expression levels of *F3′5′H_2*, 45 days old plants of *C. cajan* and *C. platycarpus* were challenged at different time intervals (8 h, 24 h, 48 h, 96 h) with the second instar larvae of *H. armigera* (Figure 1B). Further, the challenged leaf tissues were used to compare *F3′5′H_2* gene expression in the two *Cajanus* spp. From our study, it was observed that *CcF3′5′H_2* depicted downregulation at the early two time points (8 and 24 h) after the initiation of herbivory and was upregulated in the two later time points (48 and 96 h) to a maximum of 3-fold (Figure 1C). However, despite showing downregulation at 8 h after herbivory, *CpF3′5′H_2* demonstrated a linear increase in the expression levels from 24 h and was upregulated up to 4.6-fold at the end of 96 h. Accordingly, it was deduced that while *C. platycarpus* depicted an early defence response; there was a delayed response in *C. cajan*. This information not only demonstrated the involvement of *CpF3′5′H_2* in the response of the wild relative to herbivory but also corroborated with earlier studies from our group towards understanding the chemical basis of resistance response in *C. platycarpus* [11]. Though there have been studies demonstrating the utility of increased expression of *F3′5′H* in plants [21,24,25], deciphering the role of genes identified during the resistance response towards herbivory has been scarce.

### 2.2. Assessment of CcF3′5′H_2 and CpF3′5′H_2 for Variation in Gene Sequence and Copy Number

**Figure 2:**
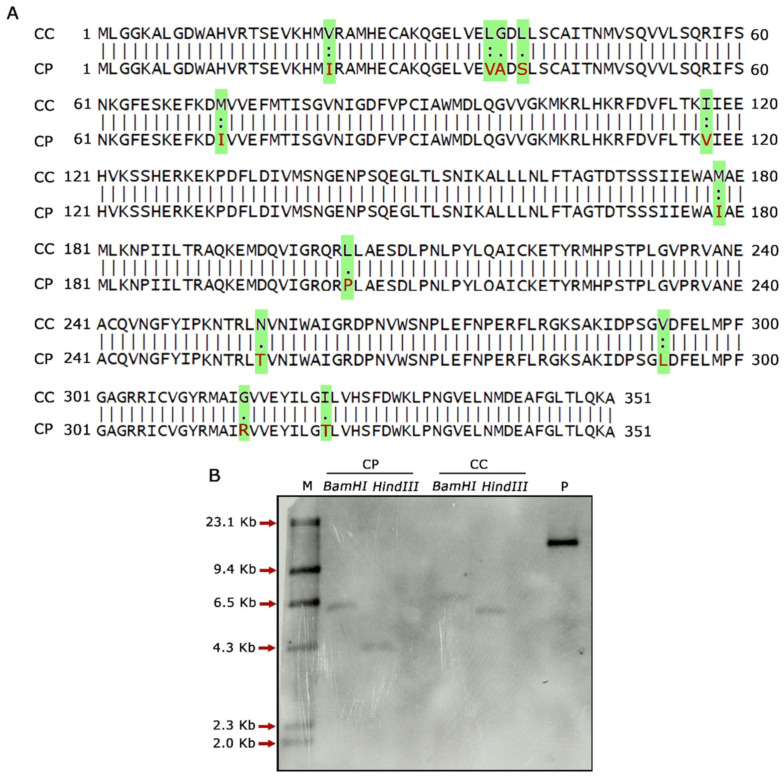
Molecular characterisation of *F3′5′H_2* gene in *C. cajan* and *C. platycarpus*. (**A**) Pairwise alignment of amino acid sequences between *F3′5′H_2* of *C. cajan* and *C. platycarpus*. Highlighted text indicates variation in amino acid sequences between the two *Cajanus* species. (**B**) Genomic Southern analysis of *F3′5′H_2* depicting gene copy number in both the *Cajanus* spp. CP, *Cajanus platycarpus*; CC, *Cajanus cajan*; M, Dig labelled marker; P, positive control-pCambia 2300 harbouring *CpF3′5′H_2* gene.

Global sequence alignment of *F3′5′H_2* in *C. cajan* and *C. platycarpus* using the Needleman–Wunsch algorithm revealed nucleotide and amino acid changes in the wild relative (Figure S1; Figure 2A), which could be apparently influencing the expression of these genes. However, an in-depth understanding of the relevance of these changes could not be divulged due to the need for domain information. Further, we wanted to check and co-relate the expression levels of *F3′5′H* to its copy number in both the genomes of *Cajanus spp*. Accordingly, genomic Southern analysis revealed that both the species harboured a single copy of the *F3′5′H_2* gene, demonstrating that the defensive response against *H. armigera* was not due to variation in gene copy number. Other factors could, therefore, be responsible for the tight regulation of *F3′5′H_2* gene in *C. platycarpus* (Figure 2B). The authenticity of the Southern analysis was confirmed by a strong hybridization signal in pCambia 2300:*CpF3′5′H_2* DNA (positive control).

**Figure 3:**
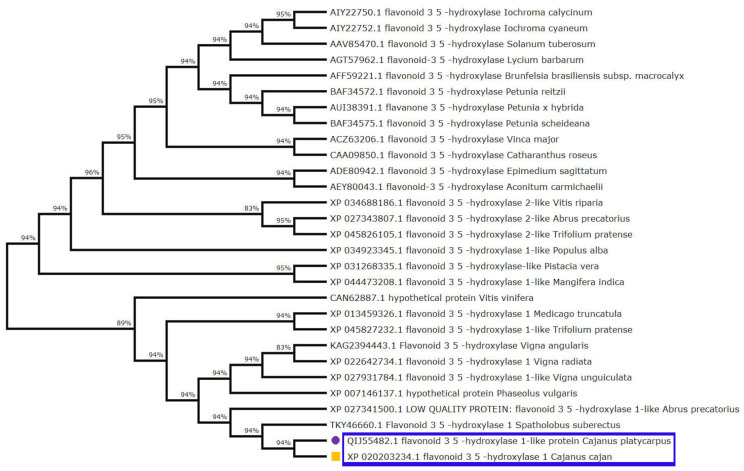
Phylogenetic tree analysis exhibiting relationship between *F3′5′H_2* from both the *Cajanus* spp. and other closely related plant species. Percentage of bootstrap values was displayed at each node, whereas branches of *C. cajan* and *C. platycarpus* were highlighted with yellow coloured square and purple coloured circle shapes, respectively.

Furthermore, multiple sequence alignment of the *F3′5′H_2* gene in both the *Cajanus* spp. under study versus other closely related plant species disclosed amino acid variations within the two species. It was seen that Gly, Ile, Val, Leu and Met of *C. cajan* were replaced by Ala/Arg, Asp/Thr, Ile/Leu, Ser and Ile of *C. platycarpus*. Further, Leu was replaced by Pro in *C. platycarpus* (Figure S2). Additionally, the diversity of the *F3′5′H_2* gene was mapped by evolutionary studies in *Cajanus* and other closely related species, which showed that 83–96% similarity was present in *Vitis riparia* followed by *Medicago trunculata*, *Trifolium pratense* and *Populus alba* (Figure 3). However, *C. cajan* and *C. platycarpus* shared 94% similarity, which depicted that the gene was distantly related to *Vigna unguiculata* and *Vitis vinifera* with 83 and 89% hit scores, respectively.

### 2.3. Assessment of Upstream Regulatory Elements of CcF3′5′H_2 and CpF3′5′H_2

**Figure 4:**
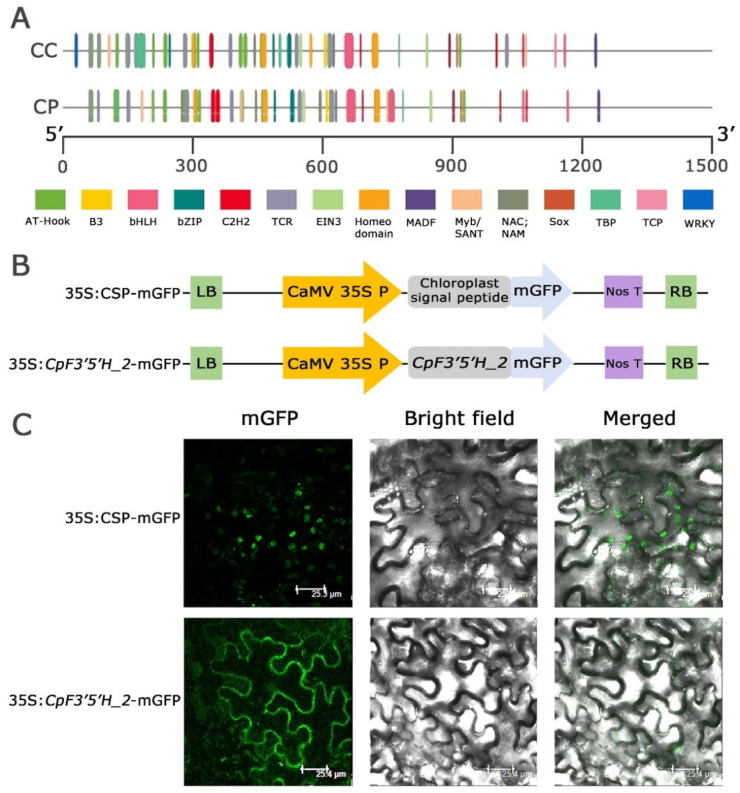
(**A**) Promoter analysis of *F3′5′H_2* gene in both the *Cajanus* spp. (**B**,**C**) Subcellular localisation of *CpF3′5′H_2* gene upon agro-infiltration into *N. benthamiana*. (**B**) Vector representations of control 35S:CSP-mGFP and 35S: *CpF3′5′H_2* -mGFP cassettes. LB: Left border; RB: Right border. (**C**) Subcellular localisation of *35S:*CSP-mGFP (control) and *CpF3′5′H_2* (gene) upon agro-infiltration into *N. benthamiana*. CSP: Chloroplast signal peptide; mGFP: modified green fluorescent protein.

Nucleotide and amino acid polymorphism, as well as expression level variation in *F3′5′H_2* from the two *Cajanus* spp., prompted us to dig deeper and understand whether these changes were due to upstream regulatory elements in the promoter regions. The promoter regions of the *F3′5′H_ 2* gene were amplified (1.3 Kb) from the two species, cloned, and the findings were analysed to compare the promoter regions (Figure S3). The results obtained from the study disclosed that transcription factors like AT hook, B3, bHLH, bZIP, C2H2, TCR, EIN3, Homeodomain, MADF, Myb/SANT, NAC, NAM, Sox and TBP were present in both the species in varied numbers while TCP and WRKY were present only in *C. cajan* (Figure 4A). Interestingly, Myb transcription factors, which are known to be stress responsive, was found in large numbers in *C. platycarpus* vis a vis *C. cajan* (2 in *C. cajan* and 4 in *C. platycarpus*). These Mybs were present at different positions within the *F3′5′H_2* gene in both species, showing deletion, insertion and 1 or 2 bp mismatches. We have found that in *C. platycarpus*, Myb was present at the 748th location, but there was a deletion at the same place in *C. cajan*. However, the 180th position of *C. platycarpus* had an insertion of a few bases in *C. cajan*. In plants, Myb transcription factors play a cardinal role in the positive or negative regulation of anthocyanin synthesis [26,27,28]. The increased number of Myb transcription factor binding sites could be the reason for the increased expression of *F3′5′H_2* during herbivory in the wild relative. 

### 2.4. Subcellular Localisation of CpF3′5′H_2 through Transient Expression in N. benthamiana

We identified the subcellular localisation of the *CpF3′5′H_2* gene by tagging to mGFP and validated the localisation using a CSP-mGFP construct (Figure 4B). GFP fluorescence of the fusion protein 35S: *CpF3′5′H_2* –mGFP was only observed in the cytoplasmic region (Figure 4C). However, mGFP tagged with a chloroplast signal peptide (CSP) as a control, showed the signal within chloroplasts, validating the cytoplasmic localisation of *CpF3′5′H_2* (Figure 4C). The results of our study were in line with earlier reports [22,29] demonstrating that the flavonoid biosynthesis pathway and enzymes involved were localised in cytoplasm [30].

### 2.5. Overexpression and Functional Validation of CpF3′5′H_2 in Tobacco for Resistance against H. armigera

The major aim of the present study was to ascertain the positive role of *CpF3′5′H_2* in managing herbivory by *H. armigera.* Before the introgression of the gene into *C. cajan,* we decided to validate it in a model plant like *N. tabacum*. Transgenic tobacco plants harbouring the *CpF3′5′H_2* gene were raised from leaf discs with the help of *Agrobacterium*-mediated genetic transformation [31]. After three rounds of kanamycin selection, the transformants were selected for further studies. Out of 25 transgenic tobacco plants, eight fully green, healthy T_0_ tobacco plants were chosen for further bio-efficacy analysis.

**Figure 5:**
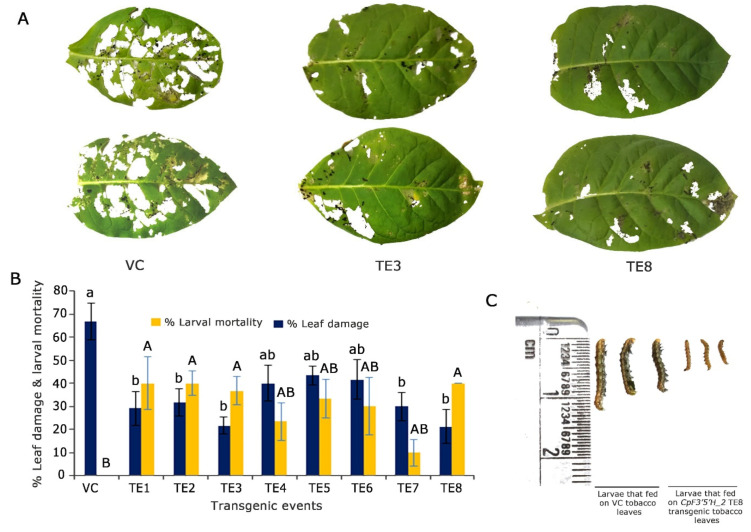
Overexpression and *in planta* validation of tobacco transgenics harbouring *CpF3′5′H_2* gene. (**A**) Bioefficacy analyses of transgenic tobacco plants harbouring *CpF3′5′H_2* and vector control (VC) plants to herbivory by 2nd instar *H. armigera*. (**B**) Graphical representation of the performance of *CpF3′5′H_2* transgenic plants and vector control plants in response to herbivore challenge [a,b and A,B denotes significance at *p* ≤ 0.05 obtained from one way ANOVA post hoc Tukey’s test]. (**C**) Morphology of larvae after feeding on vector control and *CpF3′5′H_2* overexpressed TE8 transgenic tobacco leaves.

To assess the performance of *CpF3′5′H_2* in transgenic plants, an in vitro detached leaf bioassay against the deliberate challenge of *H. armigera* was executed (Figure 5A). Transgenic plants infested with pod borer showed 30–45% leaf damage and 10–40% larval mortality. However, five plants (TE2, TE3, TE4, TE5 and TE8) that portrayed increased larval mortality of up to 40% and reduced leaf damage by up to 20% were selected for further studies. Control plants exhibited >65% leaf damage with no larval mortality (Figure 5B). Furthermore, the larvae that fed on transgenic tobacco leaves showed a reduction in growth compared to those that fed on the vector control plants (Figure 5C). Based on statistical analysis, five T_0_ putative transformants were chosen for further molecular characterisation.

Bioefficacy analysis demonstrated that *F3′5′H* from the pigeonpea wild relative *C. platycarpus* could be a potential gene for utilisation in crop improvement programmes for the management of *H. armigera.* To the best of our knowledge, this is the first report demonstrating the relevance of this gene directly during herbivory in pigeonpea. Such genes assume significance as they aid in keeping the insect at bay by the overproduction of secondary metabolites and also help the plants in better stress management [32,33]. What was more intriguing for us was the biochemical changes the overexpressing gene could be instilling in the plant.

### 2.6. Characterisation of Transgenic Tobacco for T-DNA Integration

**Figure 6:**
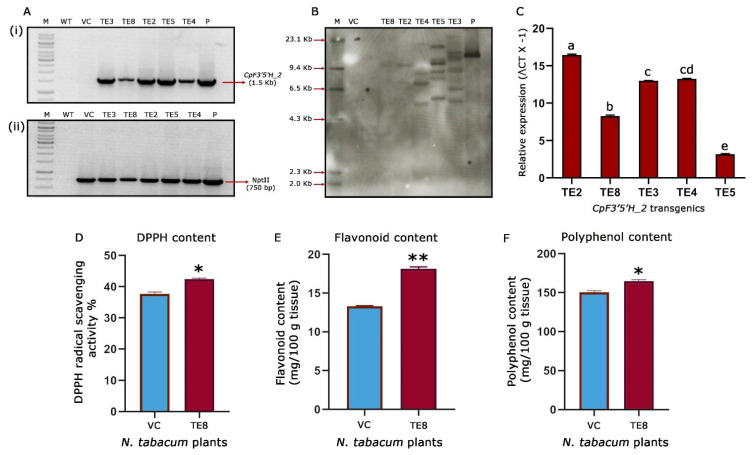
Molecular and biochemical characterisation of transgenic tobacco plants. (**A**) PCR analysis of selected transgenic, wild type and vector control plants (i) amplification of 1.5 kb *CpF3′5′H_2* gene fragment in selected transgenic tobacco plants; (ii) amplification of 750 bp *nptII* gene fragment in selected *CpF3′5′H_2* transgenic tobacco plants. P, Positive control (pCambia2300 harbouring *CpF3′5′H_2*); WT, wild type plant DNA; VC, vector control plant DNA; M, DNA marker; lanes 4–8: *CpF3′5′H_2* tobacco transgenics. (**B**) Genomic Southern analysis of *CpF3′5′H_2* tobacco transgenics. M, Dig labelled marker; VC, DNA from vector control plant; Lanes 4–8: DNA from *CpF3′5′H_2* transgenic plants; P, positive control (pCambia2300 harbouring *CpF3′5′H_2*). (**C**) Expression analysis of selected *CpF3′5′H_2* transgenic events by qRT-PCR. The value of ΔCt was calculated by the differences in the Ct values of the target gene and the reference gene; L25 ribosomal protein gene was used as an internal control. [a–e denotes significance (*p* ≤ 0.05) obtained from one-way ANOVA post hoc Tukey’s HSD test]. (**D**) Free radical scavenging activity (DPPH) in the selected single copy *CpF3′5′H_2* tobacco plant TE8. (**E**) Total flavonoid content in the selected *CpF3′5′H_2* overexpressed transgenic tobacco plant TE8 and vector control plant. (**F**) Bar graph depicting total polyphenol content in transgenic and vector control tobacco plants. [*, ** represents the significant difference at *p* ≤ 0.01 and 0.0001 from unpaired Students’s *t*-test].

Based on the leads obtained by bioefficacy analysis, five (TE2, TE3, TE4, TE5 and TE8) *CpF3′5′H_2-*overexpressed tobacco transgenic plants were chosen for further molecular characterisation. Initially, PCR amplification of a 1.5 Kb gene fragment in the transgenic plants confirmed the presence of the *CpF3′5′H_2* gene in the transformants. Additionally, amplification of a 750 bp *nptII* gene fragment confirmed the presence of the marker gene. Further, PCR analysis of vector control plants harbouring an empty T-DNA was authenticated by the amplification of a 750 bp (*nptII*) fragment and absence of the amplification of 1.5 Kb gene (*CpF3′5′H_2*) fragment (Figure 6A). Southern blotting demonstrated a single copy integration in the transgenic tobacco plant TE8, whereas the other four plants (TE2, TE3, TE4 and TE5) possessed multiple copies of the gene. The authenticity of transgenic plants was confirmed by a strong hybridization signal in pCambia 2300:*CpF3′5′H_2* positive control and no signal in vector control/negative control plants (Figure 6B). This ascertained that the reduced leaf damage and increased larval mortality in TE8 were due to the effective activity of *CpF3′5′H_2* as it possessed a single copy integration of the T-DNA.

### 2.7. Expression Analysis of CpF3′5′H_2 in Transgenic Tobacco

To experimentally prove the results obtained from bioefficacy and gene integration studies, we further performed qRT-PCR analysis to assess the expression of the *CpF3′5′H_2* gene in selected transgenic tobacco plants along with L25- ribosomal protein gene as an internal control (normaliser gene). The relative fold change expression ranged between 3.15–16.43-fold in *CpF3′5′H_2*-overexpressed transgenic tobacco plants (Figure 6C). These promising results from the study corroborated with the efficacy analysis against *H. armigera*. Nevertheless, there was a categorical increase in gene expression in those plants where the number of copies integrated was more. However, almost a nine-fold increase in gene expression was observed in TE8, being a single copy event authenticated the increased larval mortality observed in this event.

### 2.8. CpF3′5′H_2 Aids in the Management of H. armigera by Improving the ROS Scavenging Ability and Increasing Total Polyphenol and Flavonoid Content in Transgenic Tobacco

Products of the phenylpropanoid pathway, especially flavonoids, are widespread and versatile secondary metabolites in plants that are involved in growth and development as well as in stress response [34,35,36]. Several studies overexpressing either flavonoid biosynthesis genes or their regulators have demonstrated the betterment of stress management in plants mostly towards abiotic stress factors [34,37,38,39,40,41]. Further, flavonoids have been considered powerful non-enzymatic antioxidants that reduce oxidative stress in plants [35]. Studies have reported better management of oxidative and drought stress in plants due to the over-accumulation of flavonols and anthocyanins [42,43,44]. In the case of herbivory, flavonoids are utilised by plants to prevent feeding and also intervene in their development and oviposition [11,45]. Hence, it was exciting to assess some of these parameters in the transgenic tobacco plants overexpressing *CpF3′5′H_2.*

Promising results obtained after the molecular characterisation of transgenic events and their expression pattern led us to evaluate the biochemical changes in the single=copy transgenic event (TE8) further. One of the foremost markers used to assess the ability of redox management in plants is the assessment of DPPH activity [12,46]. This gives us an idea about the redox scavenging ability of the plants. In the present study, it was seen that the tobacco transgenic event TE8 showed an increased redox scavenging of up to 42.4% when compared to 37.7% in the vector control tobacco plant (Figure 6D). This provided evidence for the increased ability of oxidative stress management in the *CpF3′5′H_2* overexpressed transgenic plants. These results and other analyses ascertained the integration, over-expression, downstream activity, and bioefficacy of *CpF3′5′H_2* from the pigeonpea wild relative.

One of our previous studies elaborated on the pattern of flavonoid accumulation during continued herbivory in the wild relative and their potential role in plant defence [11]. Consistent with the earlier finding it was intriguing to see the increased DPPH activity in transgenic tobacco. This meant that the scavenging ability of the transgenics had increased due to an apparent rise in the antioxidant concentration. Coherently, increased flavonoids are known to be involved in the antioxidant response for better plant resistance to various biotic stresses [11]. To assess this as well as to reason out the increased DPPH activity in the transformants, we measured the total flavonoid, and polyphenol content in *CpF3′5′H_2* overexpressed transgenic TE8 event vis a vis the vector control plants. The results revealed a significant increase in the flavonoid and polyphenol contents in TE8 compared to VC tobacco plants. While TE8 accumulated 18.1 and 164.8 mg/100 g of tissue, respectively, of flavonoids and polyphenols, the vector control accrued 13.3 and 150.4 mg/100 g tissue of the same (Figure 6E,F). These biochemical analyses thus demonstrated the effect of overexpression of *CpF3′5′H_2* on *H. armigera* by increasing the plant’s capacity to manage oxidative stress by over-accumulating flavonoids and polyphenols. This demonstrates that overexpression of cardinal branch-point enzymes of the flavonoid pathway could improve stress management by modulating total phenols and flavonoids. Studies accruing from literature have implicated increased phenol and flavonoid content to resilience against biotic stresses like brown plant hopper in rice [47], *Botrytis cinerea* and *Dothiorella gregaria* infection in poplar [48], etc. However, a comprehensive study like the present one was not performed to manage *H. armigera* this far. This represents a new strategy for increasing the ability of the plant to manage stress as well as to keep the insect at bay. However, further intricate studies need to be carried out to ascertain metabolite status, defence mechanisms and plant responses.

The present study, therefore, demonstrates the ability of transgenic tobacco to withstand herbivory by *H. armigera* due to the overexpression of *CpF3′5′H_2,* a pivotal branch point enzyme in the flavonoid biosynthesis pathway. The study is an additional evidence [11] supporting the strong chemical resistance response prevailing in the pigoenpea wild relative towards *H. armigera*. The findings increase the possibility of incorporating *CpF3′5′H_2* as a prospective candidate gene along with other strategies like RNAi or *Bt* for managing pests as devastating as *H. armigera.*

## 3. Methods and Materials

### 3.1. Gene Source

Nucleotide sequences (cDNA) of *CcF3′5′H*_2 (XP_020203234.1) from *C. cajan* and *CpF3′5′H*_2 (QIJ55482.1) from *C. platycarpus,* identified in-house during the dynamic transcriptome analyses [13] under challenging of *H. armigera* were retrieved from National Center for Biotechnology Information (NCBI) database [49] and used for the study.

### 3.2. Plant Material and Herbivore Challenge by H. armigera

Seeds of *C. platycarpus* (ICPW 068) and *C. cajan* (TTB7) were procured from ICRISAT, Hyderabad, India and UAS, GKVK, Bangalore, India, respectively. Healthy seeds were surface sterilized by initially washing thrice with sterile double-distilled water, once with 70% ethanol (Sigma-Aldrich, St. Louis, MO, USA), followed by three washes with sterile double-distilled water. The seeds were later treated with 2% NaOCl (sodium hypochlrorite) (HiMedia, Thane, Maharashtra, India) for 1 min followed by washing with sterile double-distilled water at least four times. The sterilized seeds were sown in soil and maintained under net house conditions, care was taken that the plants were not stressed prior to challenging.

*H. armigera* larvae were collected from pigeonpea-growing fields of IARI, New Delhi, India, reared on an artificial diet and maintained under controlled conditions of 25 ± 5 °C, 70 ± 10% RH and 16 h/8 h day and light photoperiod. For the herbivore challenge, five 2nd instar *H. armigera* larvae were released on 45-day-old confined plants of *C. cajan* and *C. platycarpus,* as developed and standardized earlier [13]. Leaves of challenged plants were collected at different time intervals (8 h, 24 h, 48 h, 96 h) along with control leaves (0 h). Four biological replicates were maintained for each time interval. The collected leaves were frozen in liquid nitrogen and preserved at −80 °C until further use.

### 3.3. Expression Analysis of CcF3′5′H_2 and CpF3′5′H_2 after Herbivore Challenge

#### Total RNA Isolation and cDNA Synthesis

The total RNA was extracted from 100 mg of herbivore-challenged and control leaf samples by Spectrum^TM^ total RNA isolation kit (Sigma-Aldrich, St. Louis, MO, USA). RNA was treated with DNaseI (Sigma-Aldrich, St. Louis, MO, USA) to remove DNA contamination as per the manufacturer’s instructions. RNA was later quantified using NanoDrop 2000 (Thermo Fisher Scientific, Waltham, MA, USA) Spectrophotometer and run on 0.8% agarose gel for quantity and quality assessment. Later, cDNA synthesis was carried out using 2.5 µg of total RNA (SuperScript^®^ VILO^TM^; Invitrogen, Carlsbad, CA, USA).

### 3.4. Quantitative Real-Time PCR(qRT-PCR)

The 2 selected *F3′5′H_2* isoforms identified from the in-house developed transcriptome data were used for expression analysis using qRT-PCR (AriaMx Real-Time PCR system; Agilent, CA, USA). Gene-specific primers (Table S1), along with *initiation factor 4α* (*IF4α*) as the reference gene (Table S1), were used for analyses. The qRT-PCR conditions were as follows: initial denaturation at 95 °C for 5 min, followed by 40 cycles each of 95 °C for 10 s, 15 s at 60 °C and 15 s at 72 °C. Four independent biological and two technical replicates followed by a non-template control were used in this study. Data analyses was executed by considering 0 h as the baseline and 8 h, 24 h, 48 h and 96 h as tests. The internal reference gene was used for data normalization and fold change calculation. To determine significant (*p* ≤ 0.05) differences in gene expression between the two *Cajanus* spp., two-way ANOVA with post hoc Tukey test was performed by using the MULTCOMPVIEW package in R (https://CRAN.Rproject.org/package=multcompView; accessed on 5 November 2022).

### 3.5. Copy Number Assessment of F3′5′H_2 Gene in C. cajan and C. platycarpus

In order to identify the gene copy number of *F3′5′H_2* in *C. cajan* and *C. platycarpus*, high-quality genomic DNA was isolated from young leaves of plants according to Cetyl trimethyl ammonium bromide (CTAB; Millipore Sigma, Burlington, MA, USA) method [50]. Approximately 15 μg purified genomic DNA from both the *Cajanus* spp. was digested overnight separately with *BamHI* and *HindIII* (New England Biolabs, Ipswich, MA, USA). Additionally, about 300 pg of pCambia 2300 vector consisting of *CpF3′5′H_2* gene was linearized with *HindIII* (New England Biolabs, Ipswich, MA, USA) and used as the positive control. Overnight-restricted DNA samples were electrophoretically separated on a 0.8% agarose gel in Tris-acetate EDTA (TAE; Millipore Sigma) buffer. The separated fragments were further blotted onto a positively charged nylon membrane (Millipore Sigma) and hybridized with a Digoxigenin (DIG)-labelled 543-bp *F3′5′H_2* probe. Membrane washing and the development of X-ray film were carried out according to the manufacturer’s instructions (Roche Holding AG, Basel, CH).

### 3.6. Multiple Sequence Alignment and Phylogenetic Analysis of F3′5′H_2 in C. cajan, C. Platycarpus and Other Closely Related Species

The conserved motifs present in *F3′5′H_2* genes from both the *Cajanus spp.* were predicted using MEME online tool (http://meme-suite.org; accessed on 10 October 2022) with default parameters. The identified F3′5′H_2 protein sequences were separately aligned using the Clustal Omega server (http://ebi.ac.uk/Tool/msa/clustalo; 12 October 2022) and visualized using BioEdit 7.2 software [51]. For phylogenetic analysis, the complete F3′5′H_2 protein sequences of *C. cajan*, *C. platycarpus*, *Phaseolus vulgaris*, *Spatholobus suberectus*, *Arbus precatorius*, *Vigna radiata*, *Trifollum pratense*, *Medicago trunculata*, *Vitis vinifera*, *Populus alba*, *Petunia scheideana* and other closely related species was retrieved from NCBI database. The protein sequences were aligned in MEGA X software using the ClustalW algorithm. Phylogenetic trees were generated using the neighbour-joining method with the following parameters: pairwise deletion and 1000 bootstrap replicates.

### 3.7. Promoter Analyses of F3′5′H_2 in C. cajan and C. platycarpus

To identify variations in the promoter regions of *F3′5′H_2* in both the *Cajanus* spp., the sequences were retrieved from NCBI genome BLAST. The upstream regulatory regions of *CpF3′5′H_2* and *CcF3′5′H_2* were amplified using primers designed from the available *C. cajan* genome (Table S1). The amplified PCR products of size 1.3 Kb from both species were cloned into the pGEM-T easy vector, and sequenced by Sanger’s method (Agri Genome Labs Pvt. Ltd., Kochi, Kerala, India). The analysis of probable *cis*-elements was carried out through PlantPAN 3.0 database [52]. The simplified graphical display of *cis*-elements was projected with the help of TBtools software [53].

### 3.8. Transient Expression and Subcellular Localisation of CpF3′5′H_2 in Nicotiana benthamiana

To access the subcellular localisation of *CpF3′5′H*_2, the full-length CDS sequence of the gene was amplified (without stop codon) from *C. platycarpus* cDNA using specific primers (Table S1), cloned into the pGEM-T easy vector and confirmed by Sanger sequencing. Further, the confirmed *CpF3′5′H*_2 CDS was sub-cloned into 5′ region of mGFP in pCambia1302 vector by using *NcoI* and *SpeI* restriction sites. For control, mGFP was tagged with a chloroplast signal peptide (CSP) sequence from *C. cajan*. CSP was amplified using specific primers from the *C. cajan* cDNA sample, cloned into pGEMT-easy vector and confirmed by Sanger sequencing. Similar to *CpF3′5′H*_2, the confirmed CSP sequence was sub-cloned into the 5′ region of mGFP in pCambia1302 vector by using the same restriction sites. pCambia1302 35S:CSP-mGFP (control) and 35S:*CpF3′5′H*_2-mGFP constructs were further digested with respective restriction enzymes to confirm the positive clones and then finally transformed into *Agrobacterium tumefaciens* (GV3101).

For Agroinfiltration [13], the respective primary, secondary and tertiary cultures were incubated at 28 °C to obtain the pure culture. The cultures were then pelleted down and re-suspended in 0.5 M MES/KOH (Sigma-Aldrich, St. Louis, MO, USA) pH 5.6, 100 mM MgCl_2_ (Sigma-Aldrich, St. Louis, MO, USA), 100 mM acetosyringone (HiMedia, Thane, MH, IND) solution and later incubated in dark for 3 h [54] up to an OD_600_ of 0.5. For transient expression, one-month-old *N. benthamiana* plants were selected, and solutions with 35S:CSP-m*GFP* and 35S:*CpF3′5′H*_2-m*GFP* were infiltrated with the help of a syringe into green, fully expanded healthy leaves. After incubation for 24 h, the leaves were observed under the confocal microscope Leica SP5 with an excitation filter of 488 nm. LAS AF lite software was used for processing confocal images.

### 3.9. Validation of CpF3′5′H_2 in the Model Plant Tobacco (Nicotiana tabacum L. cv petit Havana) through a Transgenic Approach

#### Cloning of *CpF3′5′H_2* from *C. platycarpus*

The total RNA from young and healthy leaves of *C. platycarpus* was extracted using Spectrum^TM^ total RNA isolation kit (Sigma-Aldrich, St. Louis, MO, USA). The extracted RNA was qualitatively and quantitatively analyzed by using 1.2% agarose gel and Nanodrop^®^2000 (Thermo Fisher Scientific, Waltham, USA), respectively. Around 2.5 µg RNA was used for cDNA synthesis using SuperScript™ VILO™ (Invitrogen, USA) kit. The target gene (*CpF3′5′H_2*) was amplified from this cDNA using gene-specific primers (Table S1). The PCR product was visualized on 0.8% agarose gel, and the desired gene fragment was cut and eluted for further gene cloning. The eluted PCR product was ligated into the pGEM-T easy vector (Promega, Madison, WI, USA) and transformed into *E.coli* DH5α strain. The positive colonies were sequenced (Agri Genome Labs Pvt. Ltd., Kochi, Kerala, India) to confirm the cloned product, sub-cloned into a binary vector, pCambia 2300 and transformed into *A. tumefaciens* (Gv3101).

### 3.10. A. tumefaciens-Mediated Transformation of Tobacco

For the *in planta* validation of *CpF3′5′H_2*, transgenic tobacco explants were raised with the engineered construct. For vector control, tobacco explants were transformed with an empty pCambia 2300 vector without the *CpF3′5′H_2* gene. All the steps of cutting and stabilizing leaf discs, *A. tumefaciens* infection and co-cultivation were carried out as per the standardized protocol [55]. Leaf discs were further transferred onto MS selection medium containing 0.1 mg L^−1^ NAA (Millipore Sigma, Burlington, MA, USA), 2.5 mgL^−1^ BAP (Millipore Sigma, Burlington, MA, USA), 100 mg L^−1^ kanamycin (Millipore Sigma, Burlington, MA, USA; plant selection), 500 mg L^−1^ cefotaxime (Millipore Sigma, Burlington, MA, USA) and 500 mg L^−1^ carbenicillin (Millipore Sigma, Burlington, MA, USA). To maintain growth and avoid false positives, the explants were sub-cultured after 15 days onto fresh MS selection media. After attaining a height of up to 4–5 cm, the explants were transferred to a rooting medium (½ MS media) harbouring 100 mgL^−1^ kanamycin for plant selection, 500 mg L^−1^ cefotaxime and 500 mg L^−1^ carbenicillin as antibiotics. After sufficient root formation, each plant was sub-cultured to produce three replicates. Once the plants were well established, they were transferred onto soilrite. The plants were finally shifted to the net house for hardening and analyses.

### 3.11. In-Vitro Bio-Efficacy Analysis of Tobacco Transformants to H. armigera Challenge

A detached in vitro leaf bioassay was performed to check the efficacy of *CpF3′5′H_2* overexpressed transgenic events against *H. armigera* infestation. Laboratory-reared five-second instar larvae that were previously starved for 16 h were loaded onto healthy and fully expanded leaves of 40 days old transgenic tobacco plants harbouring the *CpF3′5′H_2* gene. For comparison, vector control plants were collected, and the experiment was continued up to 96 h. The percentage of leaf damage and rate of larval mortality was recorded to judge the performance of transgenic plants. Since three clones for each transformed plant were maintained, accordingly three biological and two technical replicates were used in the experiment. For statistical analysis, a one-way ANOVA post hoc Tukey test was executed to determine significant (*p* ≤ 0.05) differences in the performance of *CpF3′5′H_2* transgenics and vector control tobacco plants by using the MULTCOMPVIEW package in R (https://CRAN.Rproject.org/package=multcompView; accessed on 9 November 2022).

### 3.12. Molecular Characterisation of Transgenic Tobacco Plants

To confirm the transgenic nature of tobacco plants, high-quality genomic DNA was isolated from transformed and vector control plants by CTAB (Millipore Sigma, Burlington, MA, USA) method and used for PCR and Southern analyses. Genomic DNA was isolated as mentioned earlier and 100 ng was used for PCR analyses using *CpF3′5′H_2* gene-specific and *nptII* gene-specific primers (Table S1). The reaction consisted of initial denaturation at 95 °C for 5 min followed by 30 cycles of denaturation at 94 °C for 1 min, annealing at respective temperatures for 30 s, extension at 72 °C for 1 min and final extension at 72 °C for 10 min. The PCR-amplified products were electrophoretically separated on 0.8% agarose (Millipore, Sigma, Burlington, MA, USA) gel. For Southern analysis, genomic DNA (15 μg) from both transgenic and vector control plants was digested overnight with *HindIII* (New England Biolabs, Ipswich, MA, USA), and all the necessary steps were carried out as mentioned earlier. DNA from pCambia 2300: *CpF3′5′H_2* (300 pg) and vector control plants were considered positive and negative controls. The DIG-labelled hybridization probe was prepared by amplifying 1.5 Kb PCR product with CaMV 35S promoter forward primer and *CpF3′5′H_2* gene reverse primer (Table S1).

### 3.13. Expression Analyses of CpF3′5′H_2 in Tobacco Transgenics

Total RNA extraction from leaves of transgenic tobacco overexpressing *CpF3′5′H_2* and cDNA synthesis were carried out as mentioned earlier. Expression analyses of the *CpF3′5′H_2* gene in tobacco transgenics were performed by qRT-PCR with gene-specific primers (Table S1) as standardized earlier. The ΔCT values of *F3′5′H_2* transgenics were calculated by subtracting the CT value of the normalizer gene L25 ribosomal protein from the CT value of the *CpF3′5′H_2* gene. The SD between the replicates was calculated, and error bars were made. The significant differences between the *CpF3′5′H_2* transgenic events were calculated by one-way ANOVA post hoc Tukey test to determine *p* ≤ 0.05 by using the MULTCOMP VIEW package in R (https://CRAN.Rproject.org/package=multcompView; accessed on 9 November 2022).

### 3.14. Biochemical Analysis of Tobacco Transgenics Overexpressing CpF3′5′H_2

#### 2,2-diphenyl-1-picrylhydrazyl (DPPH) Free Radical Scavenging Activity

About 100 mg each of leaf tissues from transgenic event (TE8) and vector control tobacco were extracted in 2 mL methanol (Sigma-Aldrich, St. Louis, MO, USA) by placing it overnight on a shaker. The extracts were later centrifuged at 10,000 rpm for 15 min. The assay was carried out following the method of Mellors and Tappel [56] by using methanolic extracts with some modifications. For the assay, 0.5 mL of the methanolic extract was added to 4 mL of DPPH solution (0.1 mM) (Sigma-Aldrich, St. Louis, MO, USA), the test tubes were inverted gently and incubated in the dark for 30 min at room temperature, following which the absorbance was measured at 517 nm. The obtained results were reported as a percentage of free radical (DPPH) scavenging activity relative to control and were calculated by following the given equation:

$$\mathrm{DPPH}\;\mathrm{radical}\;\mathrm{scavenging}\;\mathrm{activity}\;(\%)=\;\frac{A_{C\;}-A_{s\;\;}\times 100}{A_{C}}$$

where *A_c_* = Absorbance of control; *A_s_* = Absorbance of sample.

To determine the significant (*p* < 0.05, 0.01, 0.001) difference, an unpaired *t*-test was performed for radical (DPPH) scavenging activity between the control and transgenic event.

### 3.15. Estimation of Total Flavonoid Content

The estimation of total flavonoid content was followed as elaborated by Tohidi et al. [57]. Briefly, 1 mL of phenolic extract and 1 mL of distilled water were mixed in a test tube. To this, 0.5 mL of 5% NaNO_2_ (Sigma-Aldrich, St. Louis, MO, USA) was added, allowing the reaction mixture to react for 6 min. Further, 0.5 mL of 10% AlCl_3_.6H_2_O (Sigma-Aldrich, St. Louis, MO, USA) was added to the reaction mixture and again allowed for 6 min to react. Later, 4 mL of 1 M NaOH (HiMedia, Thane, Maharashtra, India) and 3 mL of distilled water were added to the reaction mixture and incubated at room temperature for 15 min. Absorbance was recorded at 510 nm and a standard curve of Quercetin (Sigma-Aldrich, St. Louis, MO, USA) was prepared. Results were expressed as mg Quercetin equivalent per 100 g tissue (mg RE/100 g tissue). To determine significant (*p* < 0.05, 0.01, and 0.001) differences, an unpaired *t*-test was performed on total flavonoid content between the vector control and transgenic plants.

### 3.16. Estimation of Total Polyphenolic Content

The total phenolic content of transgenic and vector control tobacco plants was estimated using previously frozen leaf tissues. Accordingly, phenolic samples (250 µL methanol extract) and distilled water (1 mL) were mixed in a test tube, to which Folin–Ciocalteu reagent (2N) (250 µL) (Sigma-Aldrich, St. Louis, MO, USA) was added. After 6 min, 2.5 mL 7% Na_2_CO_3_ (Sigma-Aldrich, St. Louis, MO, USA) was added. This reaction mixture was then incubated at room temperature for 90 min, and absorbance was recorded at 760 nm. A standard curve of gallic acid (Sigma-Aldrich, St. Louis, MO, USA) was prepared to calculate total phenolic content, and results were expressed as mg gallic acid equivalent per 100 g of tissue (mg GAE/100 g tissue). To determine significant (*p* < 0.05, 0.01, 0.001) differences, an unpaired *t*-test was performed on the total phenolic content between the control and transgenic event.

## Data Availability

Data available on request due to restrictions e.g., privacy or ethical.

## Supplementary Materials

The supporting information can be downloaded at: https://www.mdpi.com/article/10.3390/ijms24021755/s1.
