# Supplementary material for: Cajanus platycarpus Flavonoid 3′5′ Hydroxylase_2 (CpF3′5′H_2) Confers Resistance to Helicoverpa armigera by Modulating Total Polyphenols and Flavonoids in Transgenic Tobacco
Source: Int J Mol Sci. 2023 Jan 16;24(2):1755. doi: 10.3390/ijms24021755 (PMC9862005; doi:10.3390/ijms24021755)
Supplement: Supplementary file 1 [file ijms-24-01755-s001.zip › Table S1.docx]

| **S.No.** | **Gene name** | **Primer Sequence (5' - 3')** | **Amplicon size (bp)** |
| --- | --- | --- | --- |
| q-PCR primers (Pigeonpea and Tobacco) | | | |
| 1 | *Flavonoid 3'5' hydroxylase (F3'5'H_2)* | FP- TCTGGCTTCTCCTTTCGTTC  RP- TTACAAGGTGTGGTGGGTAAG | 113 |
| 2 | Pigeonpea *Eukaryotic initiation factor 4A-10* (*IF4α)* [ Internal control] | FP- GCCGAGATCACACAGTCTCA  RP- ACCACGAGCCAAAAGATCAG | 95 |
| 3 | Tobacco ribosomal protein L25 (Internal control) | FP- CCCCTCACCACAGAGTCTGC  RP- AAGGGTGTTGTTGTCCTCAATCTT | 50 |
| Promoter primers | | | |
| 4 | *F3'5'H_2*; Set-1 | FP- TGTGTTTGTGAAAGATTATCATCCC  RP- GCATGGCTCTTATCATGTGTTTC | 1248 |
|  | *F3'5'H_2*; Set-2 | FP- CGAATTCAAGAAGAAGACGACAAC  RP- GCATGGCTCTTATCATGTGTTTC | 730 |
| Transient expression primers | | | |
| 5 | Chloroplast signal peptide | FP- CGTAGACCATGGCTTCTCGTAGTTTGAGT  RP- TAGTACACTAGTAGTACCAGTTCCTGCTTCAG | 230 |
| 6 | *CpF3'5'H_2* | FP- AGACCATGGGAATGACTTTTGATGCATTCCTTC  RP- TAGTACACTAGTAACATGTTCATAAGCATCTGAG | 1500 |
| Stable expression primers | | | |
| 7 | *CpF3'5'H_2* | FP- GTACTAGGATCCAGAATGACTTTTGATGCATTCCTTC  RP- GCTGATGGATCCTTGTATTCAAACATGTTCATAAGCATC | 1500 |
| Tobacco PCR primers | | | |
| 8 | *CpF3'5'H_2* | 35Spromoter FP- CTGAAATCACCAGTCTCTCTCTAC  *CpF3'5'H_2* RP- TTACAAGGTGTGGTGGGTAAG | 1500 |
|  | *Neomycin phosphotransferase II* (*nptII*) | FP- CCGGAATTCATGATTGAACAA  RP- CCCAAGCTTCAGAAGAACTC | 750 |
| Primers used for gene copy number analysis (Pigeonpea and Tobacco) | | | |
| 9 | *F3'5'H_2* | FP- GGCACAGACACATCATCTAGC  RP- GAGTCCAAAGGCCTCATCC | 543 |
