# Supplementary figures and images for: Cajanus platycarpus Flavonoid 3′5′ Hydroxylase_2 (CpF3′5′H_2) Confers Resistance to Helicoverpa armigera by Modulating Total Polyphenols and Flavonoids in Transgenic Tobacco
Source: Int J Mol Sci. 2023 Jan 16;24(2):1755. doi: 10.3390/ijms24021755 (PMC9862005; doi:10.3390/ijms24021755)

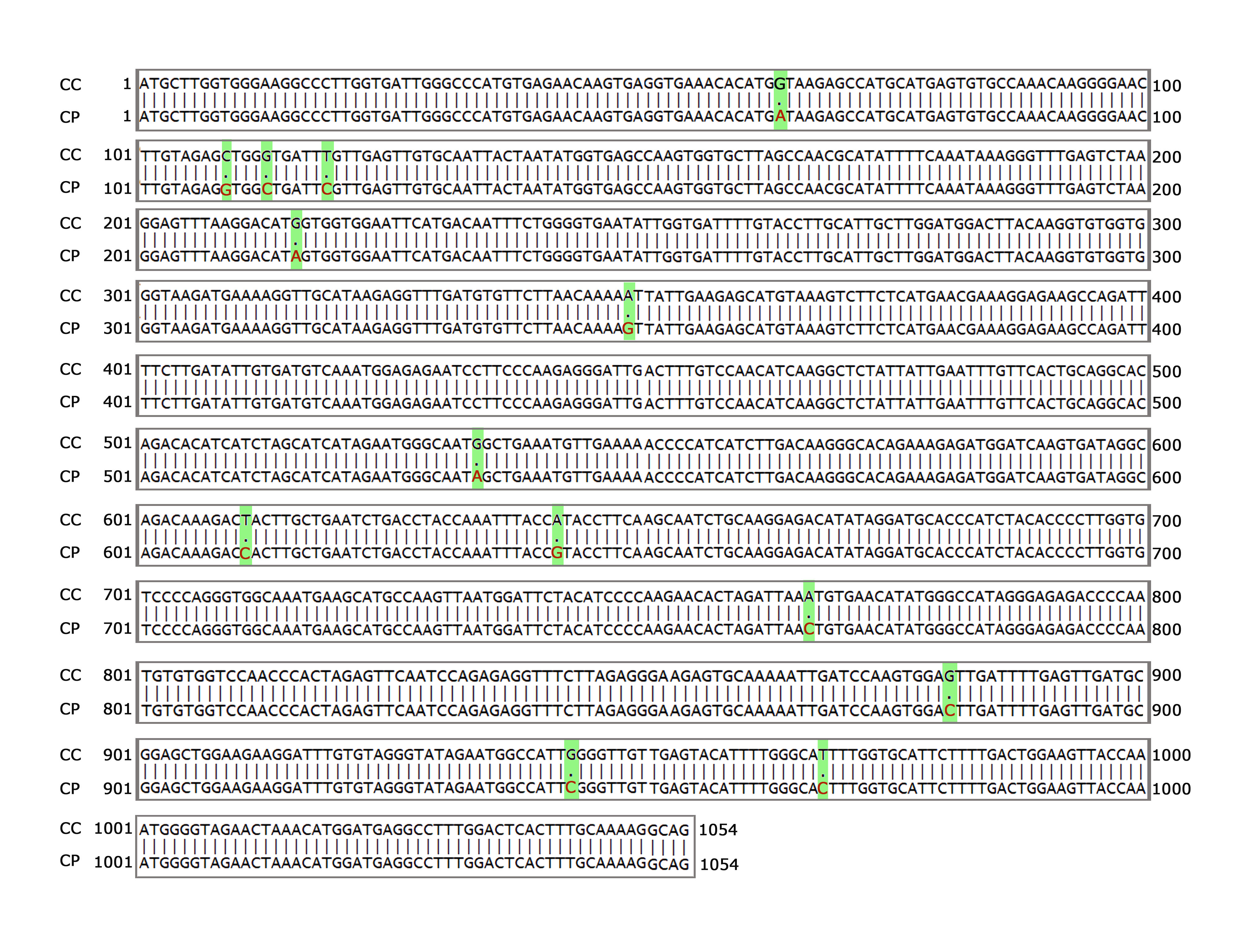

Supplement: Supplementary file 1 [file ijms-24-01755-s001.zip › Figure S1.JPG]

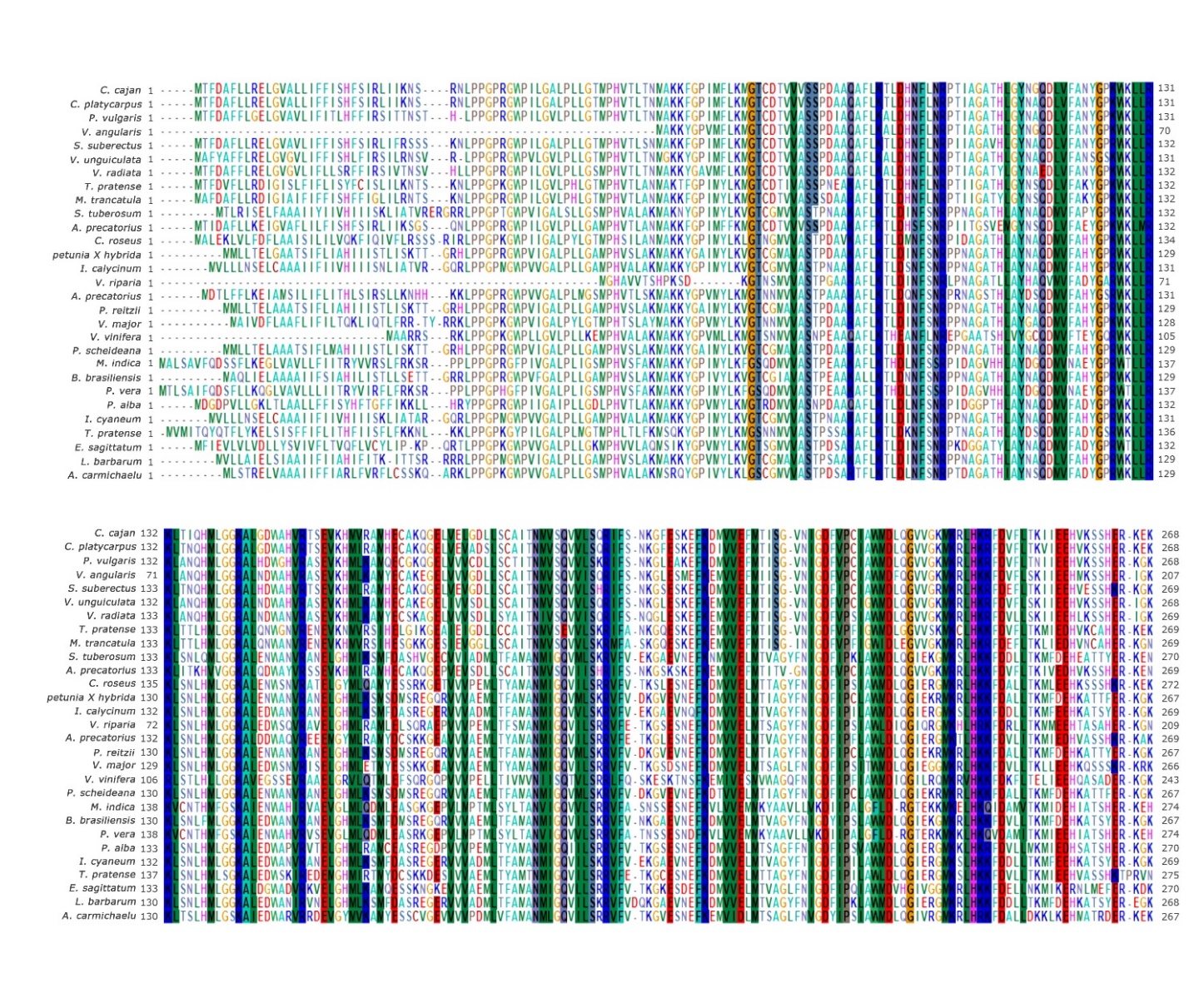

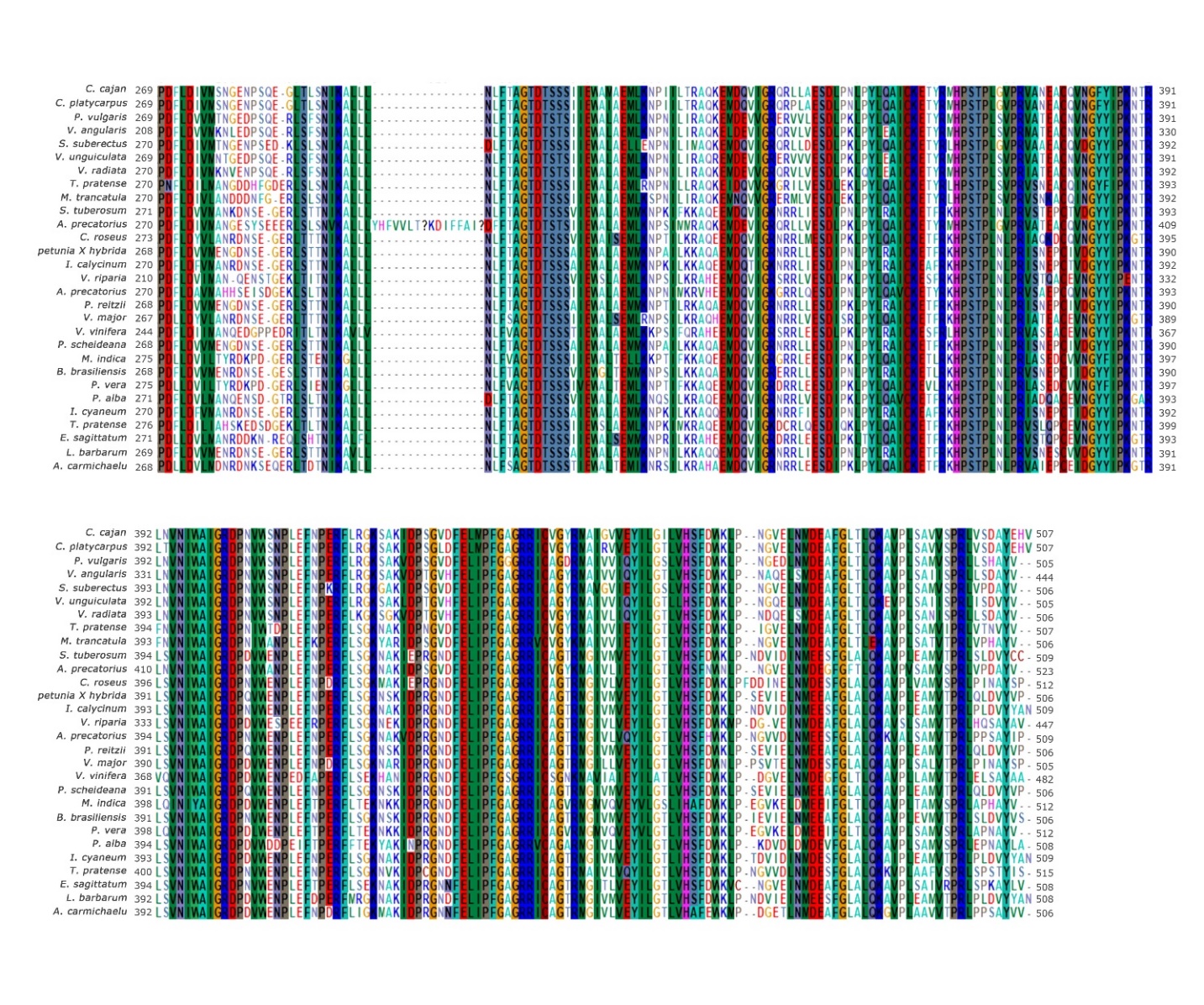

Supplement: Supplementary file 1 [file ijms-24-01755-s001.zip › Figure S2.docx]

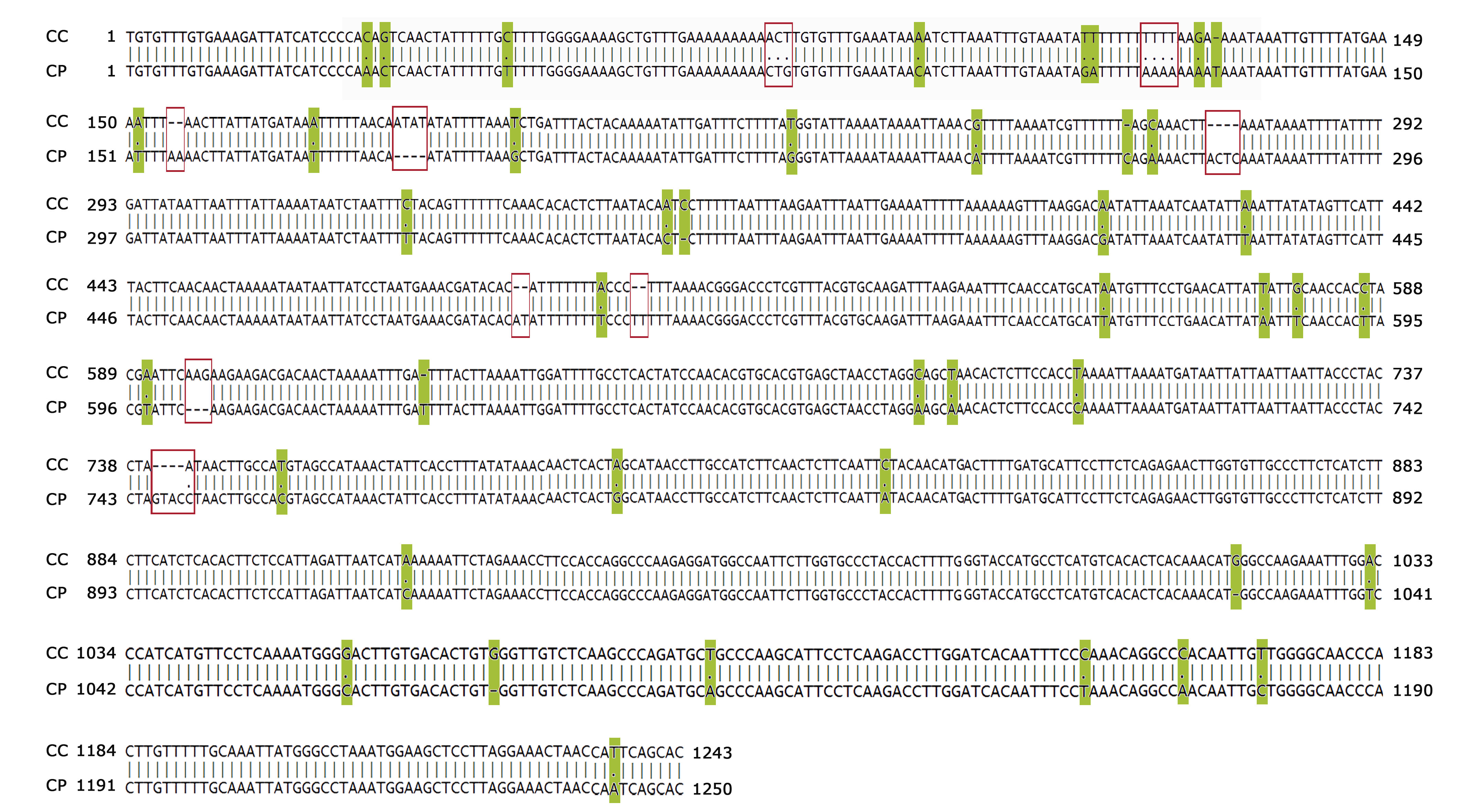

Supplement: Supplementary file 1 [file ijms-24-01755-s001.zip › Figure S3.JPG]
